# Development and validation of nomograms to predict the survival probability and occurrence of a second primary malignancy of male breast cancer patients: a population-based analysis

**DOI:** 10.3389/fonc.2023.1076997

**Published:** 2023-04-20

**Authors:** Haowei Huang, Zhuoran Li, Zhisheng Huang, Lang Huang, Wei Liu, Guolong Liu, Yuzhen Mo

**Affiliations:** ^1^ Department of Radiotherapy, Guangzhou Red Cross Hospital of Jinan University, Guangzhou, Guangdong, China; ^2^ Department of Radiology, Guangzhou Red Cross Hospital of Jinan University, Guangzhou, Guangdong, China; ^3^ Department of Rehabilitation, Guangzhou Hospital of Integrated Traditional Chinese and Western Medicine, Guangzhou, Guangdong, China; ^4^ Department of General Office, Guangdong Provincial Hospital of Occupational Disease Prevention and Treatment, Guangzhou, Guangdong, China; ^5^ Department of Breast, Guangzhou Red Cross Hospital, Guangzhou Red Cross Hospital of Jinan University, Guangzhou, Guangdong, China; ^6^ Department of Medical Oncology, Guangzhou First People’s Hospital, Jinan University, Guangzhou, Guangdong, China; ^7^ Department of Medical Oncology, Guangzhou First People’s Hospital, School of Medicine, South China University of Technology, Guangzhou, Guangdong, China

**Keywords:** male breast cancer, second primary malignancy, prognosis, survival probability, nomogram

## Abstract

**Background:**

Male breast cancer (MBC) is rare, which has restricted prospective research among MBC patients. With effective treatments, the prognosis of MBC patients has improved and developing a second primary malignancy (SPM) has become a life-threatening event for MBC survivors. However, few studies have focused on the prognosis of MBC patients and looked into the SPM issue in MBC survivors.

**Method:**

We reviewed MBC patients diagnosed between 1990 and 2016 from the latest Surveillance, Epidemiology, and End Results (SEER) Plus database. Competing risk models and nomograms were conducted for predicting the risk of cancer-specific death and SPM occurrence. C-indexes, calibration curves, ROC curves, and decision curve analysis (DCA) curves were applied for validation.

**Result:**

A total of 1,843 MBC patients with complete information were finally enrolled and 60 (3.26%) had developed an SPM. Prostate cancer (40%) was the most common SPM. The median OS of all the enrolled patients was 102.41 months, while the median latency from the initial MBC diagnosis to the subsequent diagnosis of SPM was 67.2 months. The patients who suffered from an SPM shared a longer OS than those patients with only one MBC (*p* = 0.027). The patients were randomly divided into the development cohort and the validation cohort (at a ratio of 7:3). The Fine and Gray competing risk model was used to identify the risk factors. Two nomograms were constructed and validated to predict the 5-year, 8-year, and 10-year survival probability of MBC patients, both of which had good performance in the C-index, ROC curves, calibration plots, and DCA curves, showing the ideal discrimination capability and predictive value clinically. Furthermore, we, for the first time, constructed a nomogram based on the competing risk model to predict the 5-year, 8-year, and 10-year probability of developing an SPM in MBC survivors, which also showed good discrimination, calibration, and clinical effectiveness.

**Conclusion:**

We, for the first time, included treatment information and clinical parameters to construct a nomogram to predict not only the survival probability of MBC patients but also the probability of developing an SPM in MBC survivors, which were helpful in individual risk estimation, patient follow-up, and counseling in MBC patients.

## Introduction

Breast cancer is relatively uncommon in men. Approximately 2,000 men are diagnosed with breast cancer annually in the USA, accounting for 1% of all new breast cancer patients and 0.03% of all new malignant diseases in men (1). Male breast cancer (MBC) has a similar mortality rate to female breast cancer at 17% (2). Mortality rates in Europe remained fairly stable, but the USA indicated an increase in incidence (3, 4). This trend could result from an increase in longevity in the population, since age is the major determinant of risk for most solid tumors. The incidence of MBC had a similar increasing rate with that of female breast cancer, which is probably related to the popularity of mammography screening (5, 6). However, it was shown that the prognosis of MBC patients was worse than that of female breast cancer patients (7–9). Similar to female breast cancer, the incidence of MBC also has regional differences, which is higher in North America and Europe and lower in Asia (10). The majority of MBCs do not have specific risk factors, and some small-sample studies showed that a high level of estrogen and an imbalance of hormones may contribute to the development of MBC (11–13). Genetic factors may also have a possible connection to MBC, and BRCA2 mutations appear to be the strongest risk factor for breast cancer in men with a lifetime risk of 7%, which is approximately 80 times more than the general population (14).

The rarity of MBC has restricted prospective studies on it. Principles of treatments of MBC are derived largely from randomized trials carried out in women (15, 16). Ninety percent of MBCs are estrogen-receptor-positive; tamoxifen is the standard adjuvant therapy, and some individuals could also benefit from chemotherapy. Hormonal therapy is the main treatment for metastatic disease (17), while chemotherapy can also provide palliation (10). In addition, advances in early screening and treatments have caused a considerable proportion of MBC survivors. For some survivors, second primary malignancy (SPM) is one of the most potentially life-threatening outcomes (18). At present, no research has focused on the SPM in MBC survivors, and the prediction models of developing an SPM in MBC patients have not been provided. In this study, we developed two nomogram models to predict the survival probability of MBC patients using the competing risk method. Furthermore, we built an additional nomogram to predict the probability of an MBC survivor developing an SPM.

## Method

### Data sources and population selection

The data of the present research were obtained from the latest Surveillance, Epidemiology, and End Results (SEER) Plus database (SEER 9 Registries data, with additional treatment information, Nov 2021 sub). The SEER database is an authoritative source of information on cancer, covering approximately 34.6% of the population in the USA. The records of male patients diagnosed with breast carcinoma between 1990 and 2016 were extracted using the SEER*Stat software (version 8.4.1), ensuring long-term follow-up of at least 5 years to estimate the risk of developing a second primary cancer. The International Classification of Diseases for Oncology third edition (ICD-O-3) was used to identify breast malignancy by site code C50 (including C50.1 to C50.9). The three key variables “year of diagnosis”, “sequence number”, and “total number of in situ/malignant tumors for patient” of the SEER Plus database were used to determine the status of SPM. Cases that were diagnosed as synchronous cancers occurring as SPM within 2 months after initial diagnosis or those in which the breast malignancy was not the patients’ first primary malignancy were excluded. The inclusion criteria were as follows: (1) male breast malignancy was the only or the first primary malignancy; (2) histological diagnosis confirming the existence of breast malignancy; and (3) under treatment and the follow-up data were available. The exclusion criteria were as follows: (1) incomplete cases with missing information on important variables; (2) the SPM (if any) data were incomplete; (3) initially diagnosed with distant metastasis; and (4) synchronous cancers. The flowchart of case selection is shown in **Supplementary Figure 1**.

### Variable declaration and outcome

A total of 1,843 MBC patients were involved in this study. Variables such as age, race, marital status, year of diagnosis, sequence number, total number of in *situ*/malignant tumors for patient, histological type, tumor grade, TMN stage, surgery performance, radiotherapy performance, chemotherapy performance, months from diagnosis to treatment, the hormone receptor (HR) status, HER2 status, survival time, and cause of death were extracted. Age was regrouped into six groups (<45, 45–55, 55–65, 65–75, 75–85, and 85+). Race was regrouped into white, black, and other. Marital status included married, single, and divorced. Histological type was divided into infiltrating duct, adenocarcinoma, and other by the SEER Plus database. The HR status was classified as HR positive [estrogen receptor (ER) and/or progesterone receptor (PR) was positive] and HR negative (both ER and PR were negative). TMN stage was adjusted to the 6th AJCC staging edition by the SEER Plus database in the additional analysis. The site and the diagnosis time of the SPM were recorded. Overall survival (OS) refers to the time from the initial cancer diagnosis to cancer-specific death.

### Study design and methods

The cumulative incidence of cancer-specific death and the occurrence of SPM were calculated based on the Fine and Gray competing risk model. The Kaplan–Meier method was constructed to estimate the difference in OS between MBC survivors with and without an SPM. The entire cohort was randomly divided into a development cohort (70%) and a validation cohort (30%) for the development and validation for the competing risk nomogram. Standardized mean differences (SMDs) were used to assess distributional differences in the baseline variables between the development and validation cohorts. As HER2 status is known to be tested after 2010, and HER2 status should be routinely diagnosed clearly in breast cancer patients nowadays, sensitivity analyses were carried out excluding those MBC patients whose HER2 was unknown or whose diagnosis was made prior to 2010.

### Variable selection

The univariate and multivariate Cox regression analyses were firstly performed to identify variables that significantly affected the breast cancer-specific survival and occurrence of SPM. However, applying only univariate and multivariate Cox regression analyses was inadequate, because aside from the primary tumor, there were other factors that might threaten the patients’ lives, such as accidents and infectious or other serious diseases. As a result, death due to other causes acted as a competing risk event to death due to a specific cancer. Hence, the Cox proportional hazards model might overestimate the incidence rate of the outcome with the passage of time. Similarly, death due to primary breast cancer or other causes also acted as a competing event for the MBC patients to develop an SPM—only those cured from MBC could have the probability of developing an SPM during their long survival time. In this study, the additional Fine and Gray competing risk analysis was applied to compare the association among different causes of death with a competing risk framework: death due to breast cancer or death due to other causes. Then, as for the occurrence of SPM in MBC survivors, the Fine and Gray method was also applied: death due to primary breast cancer or other causes was the competing event in the development of an SPM.

### Competing risk nomogram construction and evaluation

In order to help clinicians predict the survival probability of MBC patients and their individual probability to develop an SPM, nomograms were established based on the multivariate competing risk models. Next, we identified low-risk and high-risk survivors by calculating the 50th quantiles of total points of the nomograms and compared the difference of their survival time. Validation of these nomograms was performed by calculating the concordance index (C-index) and plotting calibration curves by a bootstrapping method with 1,000 resamples. Furthermore, the receiver operating characteristic (ROC) curves were drawn to estimate the predictive value by calculating the area under the ROC curves (AUCs). Meanwhile, decision curve analyses (DCAs) were conducted to show the clinical effectiveness of the nomogram models.

### Statistical analyses

All analyses were performed using R software (version 4.21, https://www.r-project.org/). Significance level was set as *p* < 0.05.

## Results

### Patient characteristics

**Figure 1:**
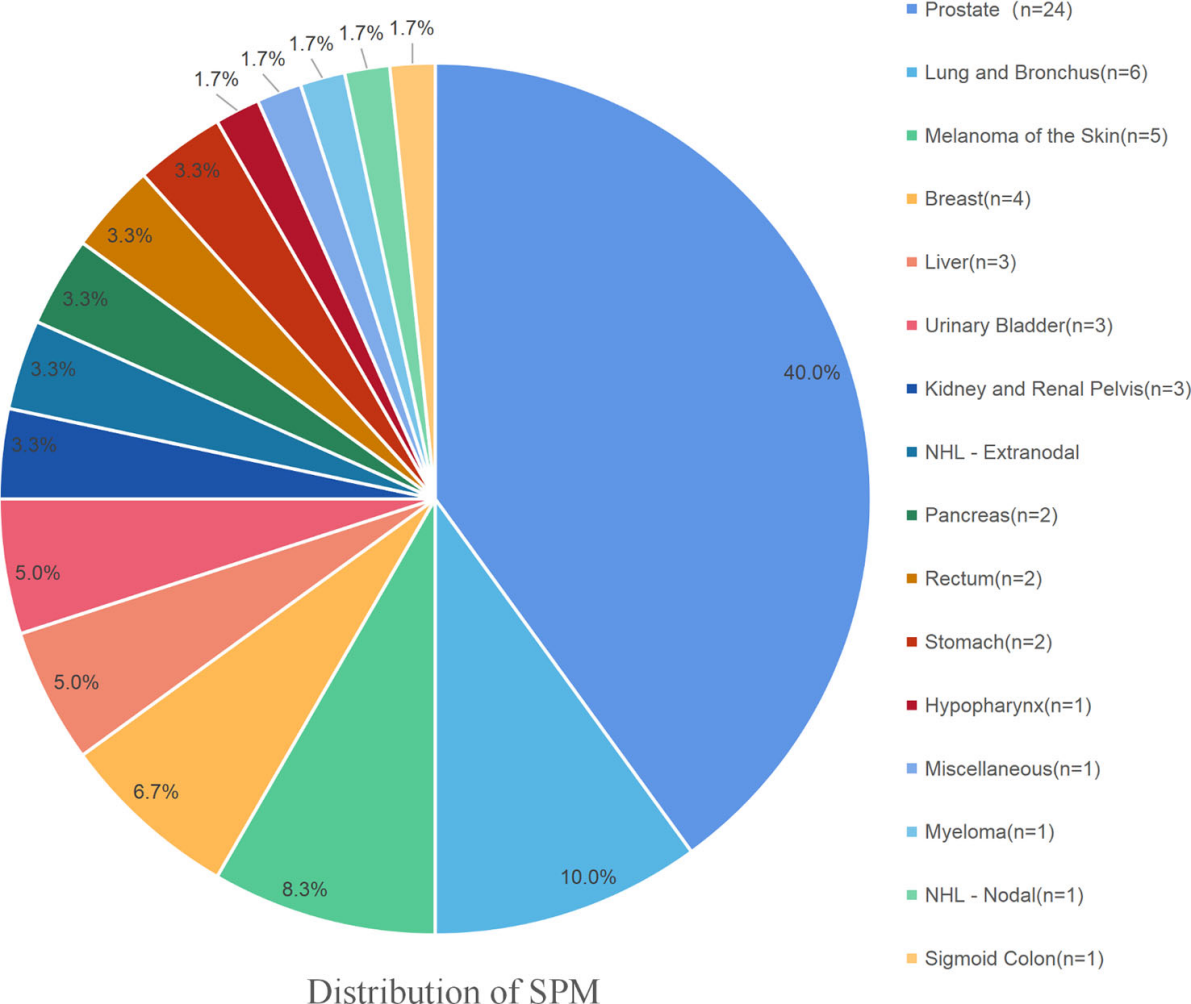
The detailed distribution of the SPMs among MBC survivors. Prostate cancer represented 24 (40%) of all SPMs, followed by lung and bronchus at 6 (10.0%), melanoma of the skin at 5 (8.3%), the secondary breast cancer at 4 (6.7%), liver at 3 (5.0%), urinary bladder at 3 (5.0%), kidney and renal pelvis at 2 (3.3%), NHL at 2 (3.3%), pancreas at 2 (3.3%), rectum at 2 (3.3%), and stomach at 2 (3.3%).

A total of 1,843 MBC patients, who were initially diagnosed between 1990 and 2016, were finally enrolled in the present study. Among these MBC patients, 60 (3.26%) developed at least one SPM. A total of 339 (18.39%) patients died from MBC, and 707 (38.4%) patients died from other causes. Among those survivors who suffered from an SPM, prostate cancer represented 24 (40%) of all SPMs, followed by lung and bronchus cancer at 6 (10.0%), melanoma of the skin at 5 (8.3%), secondary breast cancer at 4 (6.7%), liver cancer at 3 (5.0%), urinary bladder cancer at 3 (5.0%), kidney and renal pelvis cancer at 2 (3.3%), NHL at 2 (3.3%), pancreas cancer at 2 (3.3%), rectal cancer at 2 (3.3%), and stomach cancer at 2 (3.3%). The SPM details of these MBC survivors are shown in **Figure 1**. The median OS of all the enrolled patients was 102.41 months. The median latency from diagnosis of initial breast primary cancer to subsequent diagnosis of the SPM was 67.2 months. The detailed information of these MBC patients is summarized in **Tables 1**, **2**.

**Table 1:** Patient characteristics and clinicopathological variables.

Variables	Total	Cohort		*p*-value
		Development	Validation	
***N***	**1,843**	1,291	552	
**Survival months**		102.44 ± 68.48	102.37 ± 68.52	0.985
**Age**		66.86 ± 12.57	66.89 ± 12.52	0.994
**Age group**				0.962
<45	80	58 (4.49%)	22 (3.99%)	
45–55	226	154 (11.93%)	72 (13.04%)	
55–65	458	323 (25.02%)	135 (24.46%)	
65–75	527	366 (28.35%)	161 (29.17%)	
75–85	425	302 (23.39%)	123 (22.28%)	
85+	127	88 (6.82%)	39 (7.07%)	
**Race**				0.681
White	1,573	1,106 (85.67%)	467 (84.60%)	
Black	164	110 (8.52%)	54 (9.78%)	
Other	106	75 (5.81%)	31 (5.62%)	
**Marital status**				0.352
Married	1,331	937 (72.58%)	394 (71.38%)	
Single	389	263 (20.37%)	126 (22.83%)	
Divorced	123	91 (7.05%)	32 (5.80%)	
**Tumor grade**				0.207
Grade I	242	163 (12.63%)	79 (14.31%)	
Grade II	944	649 (50.27%)	295 (53.44%)	
Grade III	636	465 (36.02%)	171 (30.98%)	
Grade IV	21	14 (1.08%)	7 (1.27%)	
**Histological type**				
Infiltrating duct	1,634	1,148 (88.92%)	486 (88.04%)	0.763
Adenocarcinoma	112	75 (5.81%)	37 (6.70%)	
Other	97	68 (5.27%)	29 (5.25%)	
**TMN stage**				0.952
0	2	2 (0.15%)	0 (0.00%)	
I	645	446 (34.55%)	199 (36.05%)	
IIA	596	419 (32.46%)	177 (32.07%)	
IIB	270	189 (14.64%)	81 (14.67%)	
IIIA	153	111 (8.60%)	42 (7.61%)	
IIIC	92	64 (4.96%)	28 (5.07%)	
IIIB	85	60 (4.65%)	25 (4.53%)	
**Surgery performed**				0.847
Yes	1,821	1,276 (98.84%)	545 (98.73%)	
No	22	15 (1.16%)	7 (1.27%)	
**Radiotherapy performed**				0.976
No	1,273	892 (69.09%)	381 (69.02%)	
Yes	570	399 (30.91%)	171 (30.98%)	
**Chemotherapy performed**				0.930
No	1,158	812 (62.90%)	346 (62.68%)	
Yes	685	479 (37.10%)	206 (37.32%)	
**Duration to begin treatment**				0.281
Less than 1 month	1,060	753 (58.33%)	307 (55.62%)	
More than 1 month	783	538 (41.67%)	245 (44.38%)	
**HR status**				0.932
Positive	1,792	1,255 (97.21%)	537 (97.28%)	
Negative	51	36 (2.79%)	15 (2.72%)	
**HER2 status**				0.616
Positive	69	52 (4.03%)	17 (3.08%)	
Negative	535	373 (28.89%)	162 (29.35%)	
Borderline/Unknown	1,239	866 (67.08%)	373 (67.57%)	
**SPM occurrence**				
No	1,783	1,255 (97.21%)	528 (95.65%)	0.084
Yes	60	36 (2.79%)	24 (4.35%)	

^*^Statistically significant (p < 0.05).

**Table 2:** Patient characteristics (with or without SPM).

Variables	Total	Occurrence of SPM		*p*-value
		No	Yes	
***N***	**1,843**	1,783	60	
**Survival months**		101.87 ± 68.17	118.63 ± 75.76	0.027*
**Age**		66.84 ± 12.63	67.53 ± 10.02	0.817
**Age group**				0.160
<45	80	80 (4.49%)	0 (0.00%)	
45–55	226	218 (12.23%)	8 (13.33%)	
55–65	458	444 (24.90%)	14 (23.33%)	
65–75	527	502 (28.15%)	25 (41.67%)	
75–85	425	415 (23.28%)	10 (16.67%)	
85+	127	124 (6.95%)	3 (5.00%)	
**Race**				0.637
White	1,573	1,524 (85.47%)	49 (81.67%)	
Black	164	158 (8.86%)	6 (10.00%)	
Other	106	101 (5.66%)	5 (8.33%)	
**Marital status**				0.172
Married	1,331	1,294 (72.57%)	37 (61.67%)	
Single	389	372 (20.86%)	17 (28.33%)	
Divorced	123	117 (6.56%)	6 (10.00%)	
**Tumor grade**				0.202
Grade I	242	229 (12.84%)	13 (21.67%)	
Grade II	944	915 (51.32%)	29 (48.33%)	
Grade III	636	618 (34.66%)	18 (30.00%)	
Grade IV	21	21 (1.18%)	0 (0.00%)	
**Histological type**				
Infiltrating duct	1,634	1,583 (88.78%)	51 (85.00%)	0.538
Adenocarcinoma	112	108 (6.06%)	4 (6.67%)	
Other	97	92 (5.16%)	5 (8.33%)	
**TMN stage**				0.952
0	2	2 (0.11%)	0 (0.00%)	
I	645	624 (35.00%)	21 (35.00%)	
IIA	596	577 (32.36%)	19 (31.67%)	
IIB	270	261 (14.64%)	9 (15.00%)	
IIIA	153	146 (8.19%)	7 (11.67%)	
IIIC	92	91 (5.10%)	1 (1.67%)	
IIIB	85	82 (4.60%)	3 (5.00%)	
**Surgery performed**				0.732
Yes	1,821	1,762 (98.82%)	59 (98.33%)	
No	22	21 (1.18%)	1 (1.67%)	
**Radiotherapy performed**				0.488
No	1,273	1,234 (69.21%)	39 (65.00%)	
Yes	570	549 (30.79%)	21 (35.00%)	
**Chemotherapy performed**				0.370
No	1,158	1,117 (62.65%)	41 (68.33%)	
Yes	685	666 (37.35%)	19 (31.67%)	
**Duration to begin treatment**				0.233
Less than 1 month	1,060	1,021 (57.26%)	39 (65.00%)	
More than 1 month	783	762 (42.74%)	21 (35.00%)	
**HR status**				0.597
Positive	1,792	1,733 (97.20%)	59 (98.33%)	
Negative	51	50 (2.80%)	1 (1.67%)	
**HER2 status**				0.616
Positive	69	69 (3.87%)	0 (0.00%)	
Negative	535	524 (29.39%)	11 (18.33%)	
Borderline/Unknown	1,239	1,190 (66.74%)	49 (81.67%)	

^*^Statistically significant (p < 0.05).

### Kaplan–Meier analysis

**Figure 2:**
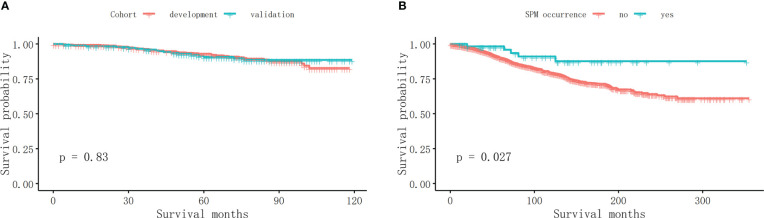
The survival analyses. **(A)** There was no significant difference in OS between the development and validation cohort (*p* = 0.83). **(B)** Those who developed an SPM have a significantly longer OS in MBC survivors (*p* = 0.027).

As is shown in **Figure 2A**, there was no significant difference in OS between the development and validation cohorts (*p* = 0.83). The OS of MBC patients who did not suffer from an SPM was 101.87 ± 68.17 months, while the OS of those who suffered from an SPM was 118.63 ± 75.76 months. Those who developed an SPM have a significantly longer OS (**Figure 2B**, *p* = 0.027).

### Univariate and multivariate Cox regression analysis with cancer-specific death

Univariate and multivariate Cox regression were applied in the development cohort to select the predictive variables for the prediction models of cancer-specific death. MBC patients whose HER2 was unknown or diagnosed prior to 2010 were excluded in the following sensitivity analyses as mentioned above. As is shown in **Table 3**, tumor grade, TMN stage, surgery, and chemotherapy were related to OS in the univariate analysis, while in the multivariate Cox regression, chemotherapy failed to show a significant relation with OS.

**Table 3:** Univariate and multivariate analysis of cancer-specific death.

Variables	Univariate analysis		Multivariate analysis	
	HR (95% CI)	*p*-value	HR (95% CI)	*p*-value
Age group				
65–75	1		1	
55–65	0.78 (0.37, 1.66)	0.5205	0.5 (0.2, 1.4)	0.182
75–85	0.84 (0.40, 1.77)	0.6561	1.8 (0.6, 5.6)	0.288
45–55	0.73 (0.26, 2.03)	0.5438	0.2 (0.0, 1.6)	0.135
85+	0.88 (0.28, 2.73)	0.8249	2.06 (0.68, 6.26)	0.2024
<45	1.56 (0.49, 5.01) 0.4551	0.4551	0.4 (0.0, 6.3)	0.481
Race				
White	1		1	
Black	1.53 (0.71, 3.29)	0.2745	1.5 (0.7, 3.3)	0.355
Other	0.84 (0.25, 2.83)	0.7745	0.6 (0.2, 1.9)	0.360
Marital status				
Married	1		1	
Single	0.80 (0.40, 1.60)	0.5307	0.8 (0.4, 1.6)	0.523
Divorced	1.42 (0.53, 3.85)	0.4870	1.0 (0.3, 2.7)	0.928
Tumor grade				
Grade II	1		1	
Grade III	2.17 (1.22, 3.87)	0.0083*	1.9 (1.1, 3.4)	0.029*
Grade I	0.66 (0.22, 1.98)	0.4601	1.0 (0.3, 3.1)	0.954
Grade IV	0.00 (0.00, Inf)	0.9891	0.0 (0.0, Inf)	0.998
Histological type				
Infiltrating duct	1		1	
Adenocarcinoma	0.73 (0.17, 3.18)	0.6791	1.2 (0.3, 5.2)	0.814
Other	0.51 (0.12, 2.18)	0.3625	0.5 (0.1, 2.0)	0.316
TMN stage				
Stage 0+I	1		1	
Stage II	2.57 (1.15, 5.74)	0.0213*	2.2 (1.0, 5.0)	0.061*
Stage III	6.33 (2.68, 14.98)	<0.0001*	5.6 (2.2, 14.6)	<0.001*
Surgery performed				
Yes	1		1	
No	5.16 (1.70, 15.68)	0.0038*	3.8 (1.4, 10.6)	0.009*
Radiotherapy performed				
No	1		1	
Yes	1.51 (0.87, 2.63)	0.1474	0.9 (0.5, 1.8)	0.870
Chemotherapy performed				
No	1		1	
Yes	2.56 (1.47, 4.48)	0.0009*	1.3 (0.6, 2.6)	0.520
Duration to begin treatment				
Less than 1 month	1		1	
More than 1 month	0.85 (0.49, 1.46)	0.5494	1.1 (0.6, 1.9)	0.765
HR status				
Positive	1		1	
Negative	0.96 (0.12, 7.63)	0.9684	0.7 (0.1, 5.8)	0.764
HER2 status				
Negative	1		1	
Positive	11.52 (0.71, 3.26)	0.2793	1.2 (0.6, 2.7)	0.603

^*^Statistically significant (p < 0.05).

### Univariate and multivariate Cox regression analysis with the occurrence of SPM

Univariate and multivariate Cox regression were also applied to select the predictive variables for the occurrence of SPM. As is shown in **Table 4**, marital status showed a significant relation with the occurrence of SPM in the univariate analysis. Moreover, in the multivariate Cox regression, age, race, tumor differentiated grade, histological type, TMN stage, chemotherapy, and the waiting time from diagnosis to begin treatment were significant.

**Table 4:** Univariate and multivariate analysis of the occurrence of SPM.

Variables	Univariate analysis		Multivariate analysis	
	HR (95% CI)	*p*-value	HR (95% CI)	*p*-value
Age group				
65–75	1.0		1.0	
55–65	0.62 (0.15, 2.54)	0.5103	0.1 (0.0, 0.6)	0.009*
75–85	0.20 (0.02, 1.71)	0.1429	17,323.8 (1570.8, 191059.2)	<0.001*
45–55	0.47 (0.05, 3.94)	0.4831	0.0 (0.0, 0.0)	<0.001*
85+	0.00 (0.00, Inf)	0.9921	0.7 (0.0, Inf)	1.000
<45	0.00 (0.00, Inf)	0.9939	0.6 (0.0, Inf)	1.000
Race				
White	1.0		1.0	
Black	1.64 (0.35, 7.77)	0.5310	Inf (Inf, Inf)	<0.001*
Other	0.00 (0.00, Inf)	0.9928	349.3 (0.0, Inf)	1.000
Marital status				
Married	1.0		1.0	
Single	3.10 (0.77, 12.57)	0.1127	45.9 (10.8, 195.3)	<0.001*
Divorced	9.04 (1.95, 42.02)	0.0050*	0.0 (0.0, 0.0)	<0.001*
Tumor grade				
Grade II	1.0		1.0	
Grade III	0.40 (0.08, 1.95)	0.2575	Inf (Inf, Inf)	<0.001*
Grade I	1.07 (0.22, 5.24)	0.9355	Inf (Inf, Inf)	<0.001*
Grade IV	0.00 (0.00, Inf)	0.9935	1.4 (0.0, Inf)	1.000
Histological type				
Infiltrating duct	1.0		1.0	
Adenocarcinoma	2.55 (0.31, 21.18)	0.3853	0.0 (0.0, 0.0)	<0.001*
Other	3.69 (0.76, 18.01)	0.1068	Inf (4,969,507.8, Inf)	<0.001*
TMN stage				
I	1.0		1.0	
IIA	0.51 (0.05, 5.64)	0.5811	Inf (Inf, Inf)	<0.001*
IIB	4.95 (0.94, 25.96)	0.0585	1957392.5 (318,862.9, Inf)	<0.001*
IIIA	5.56 (0.76, 40.72)	0.0915	1.5 (0.1, 16.9)	0.727
IIIC	0.00 (0.00, Inf)	0.9906	1.4 (0.0, Inf)	1.000
IIIB	3.57 (0.31, 40.68)	0.3051	0.3 (0.0, 4.9)	0.404
0	0.00 (0.00, Inf)	0.9983	0.8 (0.0, Inf)	1.000
Surgery performed				
Yes	1.0		1.0	
No	0.00 (0.00, Inf)	0.9931	0.7 (0.0, Inf)	1.000
Radiotherapy performed				
No	1.0		1.0	
Yes	1.14 (0.33, 3.94)	0.8360	0.0 (0.0, 0.0)	<0.001*
Chemotherapy performed				
No	1.0		1.0	
Yes	1.92 (0.58, 6.37)	0.2856	2.8 (0.5, 15.1)	0.239
Duration to begin treatment				
Less than 1 month	1.0		1.0	
More than 1 month	3.30 (0.71, 15.42)	0.1286	6,062,810.1 (987,643.0, Inf)	<0.001*
HR status				
Positive	1.0		1.0	
Negative	0.00 (0.00, Inf)	0.9909	0.5 (0.0, Inf)	1.000
HER2 status				
Negative	1.0		1.0	
Positive	0.00 (0.00, Inf)	0.9903	1.0 (0.0, Inf)	1.000

^*^Statistically significant (p < 0.05).

### Fine and Gray competing risk models

The Fine and Gray method was used to estimate the risk predictors for cancer-specific death and the occurrence of SPM. The results of the characteristics are provided in **Table 5**. Age, race, marital status, histological type, TMN stage, therapy, the waiting time from diagnosis to begin treatment, HR status, and HER2 status were the significant risk factors for both cancer-specific death and the development of an SPM.

**Table 5:** Risk factors associated with cancer-specific death and occurrence of SPM.

Variables	Cancer-specific death		Occurrence of SPM	
	SHR	*p*-value	SHR	*p*-value
Age group				
65–75	ref		ref	
55–65	1.0137	<0.0001*	0.7516	<0.0001*
75–85	2.3679	<0.0001*	0.7861	<0.0001*
45–55	1.8132	<0.0001*	0.8458	<0.0001*
85+	0.547	<0.0001*	0.8998	<0.0001*
<45	6.5540	<0.0001*	0.9159	<0.0001*
Race				
White	ref		ref	
Black	1.7756	<0.0001*	1.0583	<0.0001*
Other	0.5697	<0.0001*	0.9682	<0.0001*
Marital status				
Married	ref		ref	
Single	0.5560	<0.0001*	0.8393	<0.0001*
Divorced	0.5677	<0.0001*	1.0487	<0.0001*
Tumor grade				
Grade II	ref		ref	
Grade III	1.5597	<0.0001*	1.0574	<0.0001*
Grade I	0.1559	<0.0001*	0.8183	<0.0001*
Grade IV	0.0153	<0.0001*	0.6641	<0.0001*
Histological type				
Infiltrating duct	ref		ref	
Adenocarcinoma	0.0486	<0.0001*	1.5327	<0.0001*
Other	0.3203	<0.0001*	1.0089	<0.0001*
TMN stage				
Stage 0+I	ref		ref	
Stage II	0.0912	<0.0001*	0.6253	<0.0001*
Stage III	4.0035	<0.0001*	0.6135	<0.0001*
Surgery performed				
Yes	ref		ref	
No	2.9478	<0.0001*	1.1238	<0.0001*
Radiotherapy performed				
No	ref		ref	
Yes	0.6601	<0.0001*	1.1854	<0.0001*
Chemotherapy performed				
No	ref		ref	
Yes	1.9436	<0.0001*	0.9004	<0.0001*
Duration to begin treatment				
Less than 1 month	ref		ref	
More than 1 month	0.7742	<0.0001*	0.9765	<0.0001*
HR status				
Positive	ref		ref	
Negative	0.0939	<0.0001*	0.9360	<0.0001*
HER2 status				
Negative	ref		ref	
Positive	1.5371	<0.0001*	1.1042	<0.0001*

^*^Statistically significant (p < 0.05).

### Nomogram construction and validation

**Figure 3:**
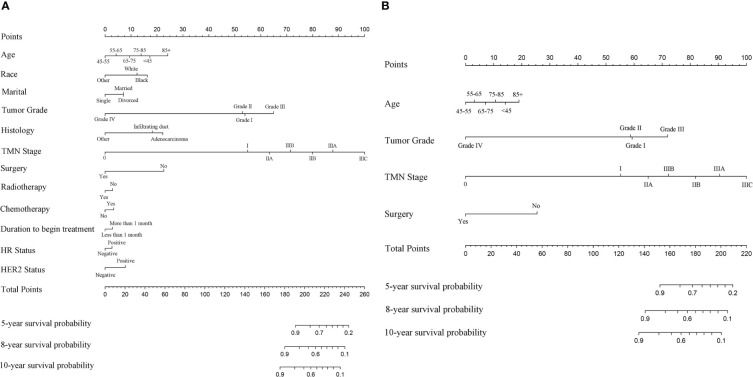
**(A)** The nomogram model 1 to predict the 5-year, 8-year, and 10-year survival probability of MBC patients based on the Fine and Gray method. **(B)** The nomogram model 2 to predict the same survival probability of MBC patients based on the multivariate Cox regression.

**Figure 4:**
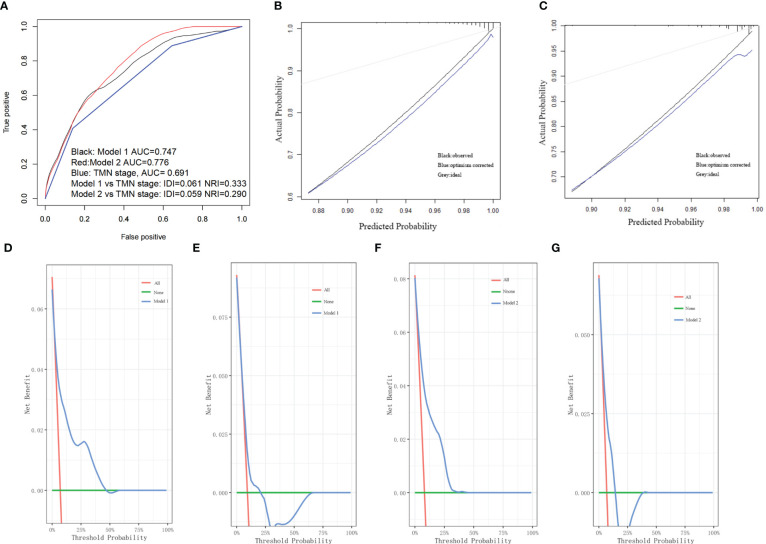
**(A)** Both model 1 and model 2 showed better predictive value than TMN stage in the ROC analyses. **(B)** The calibration curve of model 1. **(C)** The calibration curve of model 2. **(D)** The DCA of model 1 in the development cohort. **(E)** The DCA of model 1 in the validation cohort. **(F)** The DCA of model 2 in the development cohort. **(G)** The DCA of model 2 in the validation cohort.

**Figure 5:**
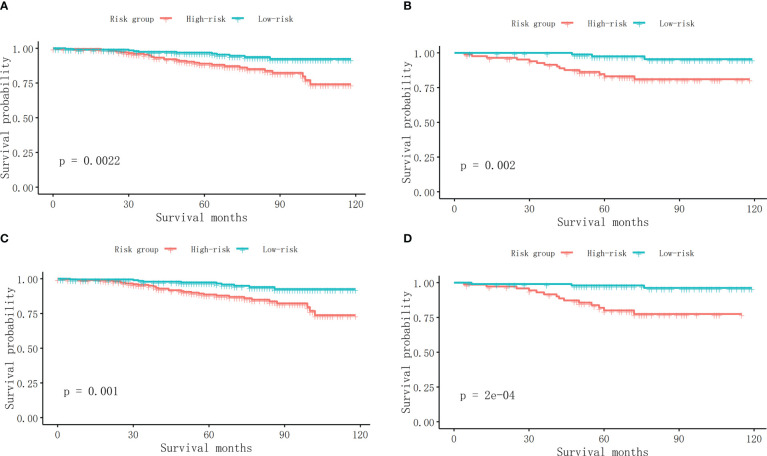
**(A)** The survival curves between different risk groups in the development cohort in model 1. **(B)** The survival curves between different risk groups in the validation cohort in model 1. **(C)** The survival curves between different risk groups in the development cohort in model 2. **(D)** The survival curves between different risk groups in the validation cohort in model 2.

The first two nomograms were established based on the previously mentioned risk factors to predict the survival probability of MBC patients. Age, race, marital status, tumor differentiated grade, histology, TMN stage, surgery, radiotherapy, chemotherapy, duration to begin treatment, HR status, and HER2 status, which were selected by the Fine and Gray method, were enrolled in nomogram model 1 to predict the 5-year, 8-year, and 10-year survival probability of MBC patients (**Figure 3A**). Meanwhile, age, tumor differentiated grade, TMN stage, and surgery, which were selected by the multivariate Cox regression, were included in nomogram model 2 (**Figure 3B**) to predict the same survival probability above. The C-index of model 1 was 0.710 in the development cohort and 0.703 in the validation cohort, while model 2 had a C-index at 0.728 in the development cohort and 0.718 at the validation cohort. Both model 1 (AUC = 0.713) and model 2 (AUC = 0.757) achieved a better predictive value than the AJCC TMN staging system (AUC = 0.689) in the ROC analysis shown in **Figure 4A**. The integrated discrimination improvement (IDI) and net reclassification improvement (NRI) between model 1 and TMN stage were 0.610 (95% CI 0.490–0.258) and 0.333 (95% CI 0.182–0.508), respectively. Meanwhile, The IDI and NRI between model 2 and TMN stage were 0.059 (95% CI 0.036–0.193) and 0.290 (95% CI 0.154–0.513), respectively. The calibration curves show that both model 1 and model 2 had good agreement between predicted probability and the observed outcome (**Figures 4B, C**). The DCA also showed that model 1 (**Figures 4D, E**) and model 2 (**Figures 4F, G**) had a good discrimination in both the development and validation cohorts. We divided the patients into a low-risk group and a high-risk group at the 50th percentile of nomogram total points and compared the difference of the survival time among these subgroups. **Figures 5A, B** show that there were significant differences in survival time between different risk groups in both the development cohort (*p* = 0.0022) and the validation cohort (*p* = 0.002), based on nomogram model 1 (**Figure 3A**). Meanwhile, **Figures 5C, D** also show the survival time difference between different risk groups in the development cohort (*p* = 0.001) and the validation cohort (*p* = 2e-04), based on nomogram model 2 (**Figure 3B**), which indicated that both of these nomogram models had a good discrimination capability for the survival probability of the MBC patients. The details of these two nomograms are shown in **Supplementary Tables 1**, **2**.

**Figure 6:**
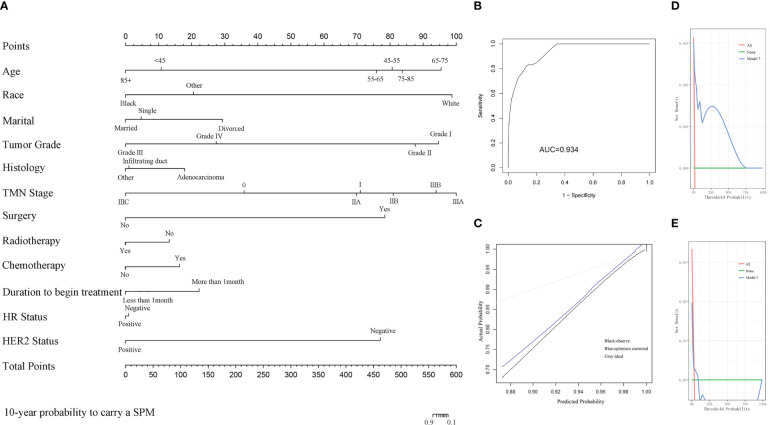
**(A)** The nomogram of model 3 for predicting the 10-year probability of MBC survivors who suffer from an SPM. **(B)** The ROC curve of model 3. **(C)** The calibration curve of model 3. **(D)** The DCA of model 3 in the development cohort. **(E)** The DCA of model 3 in the validation cohort.

An additional nomogram model 3 was established to predict the probability of MBC survivors developing an SPM within 10 years after the initial diagnosis. All of the risk factors selected by the Fine and Gray method were included in model 3 (**Figure 6A**). The C-index of model 3 was 0.909 in the development cohort and 0.494 in the validation cohort. The AUC of the ROC curve in model 3 is 0.934 (**Figure 6B**). The calibration curve is shown in **Figure 6C**. The DCA curve is shown in **Figure 6D** in the development cohort and in **Figure 6E** in the validation cohort. The details of these risk factors are shown in **Supplementary Table 3**.

## Discussion

MBC is a rare disease whose causes remain incompletely characterized and understood. Because of the limitation of large-scale randomized prospective research, MBC treatment largely follows the guidelines of female breast cancer (19). By applying sufficient therapies, such as surgery, chemotherapy, radiotherapy, endocrine therapy, targeted therapy, and immunotherapy, the prognosis of MBC survivors has improved in the past 25 years (20). With the longevity of the MBC survivors, SPM has become a life-threatening event. In the present study, we enrolled 1,843 MBC patients who were randomly divided into a development and a validation group at a ratio of 7:3. No difference was found between these two groups (**Table 1**). At present, a few studies focused on the prognosis of MBC patients. Wang et al. developed a nomogram to predict distant metastasis in MBC patients, based on univariate and multivariate logistic regression analyses, but did not focus on the probability of survival and the development of an SPM (21). Chen et al. constructed a nomogram to predict the prognosis of MBC patients based on univariate and multivariate Cox regression (22). Similar research was published by Zhang et al. (23). However, as we mentioned above, applying only Cox regression analysis was inadequate and would overestimate the risk of cancer-specific death, because aside from the primary tumor, there were other factors that might threaten their life (24), and death due to other causes actually acted as a competing event to death caused by MBC. In this study, two nomograms were constructed to predict the survival probability of MBC patients based on the Fine and Gray competing risk analysis and multivariate Cox regression, respectively, to correct this bias. Sun et al. performed a competing risk analysis in MBC patients but failed to include treatment information (25). As is shown in the present study, treatments influenced cancer-specific death and the occurrence of SPM. Different clinical circumstances with different treatment strategies might lead to different outcomes.

A few studies estimated the effect of initial treatment on the development of SPM in female breast cancer patients (26, 27), but no research has focused on the development of SPM in MBC survivors. To our knowledge, this is the first available nomogram for developing an SPM in MBC survivors in the presence of competing events.

In this study, 60 survivors developed an SPM. Prostate cancer was the most common SPM. Interestingly, previous research showed that prostate cancer was also the most common SPM in colon cancer survivors treated with colectomy (28). However, prostate cancer had a bigger portion in SPM patients than in the whole population (29). The efficiency of endocrine therapy, along with the high proportion of HR-positive status in MBC patients (17), warrants further study to clarify whether the endocrine status is related to the occurrence of the SPM. It is also worth noting that patients who suffered from an SPM shared a longer OS than those patients with only one MBC (**Figure 2**, *p* = 0.027), which indicated that the cumulative incidence of developing an SPM increased with the prolonged survival time.

Univariate and multivariate Cox regression analyses were insufficient, and in this study, we applied additional Fine and Gray competing risk analysis to show the differences among the risk factors associated with OS and the occurrence of SPM. We have constructed two nomogram models to predict the OS of the MBC patients: model 1 based on the risk factors selected by the Fine and Gray method, and model 2 based on the multivariate analysis. Both of these nomogram models achieved good C-index. Model 2 had an even better predictive value than model 1 and the TMN stage in the combined ROC analysis (**Figure 4A**). The calibration plots, the DCA curves, and the survival curves of different risk groups altogether showed that both of these models had an ideal discrimination capability and predictive value. Model 1 included more clinical details while model 2 was more simplified. According to our study, higher age at diagnosis, higher TMN stage, absence of surgery and radiotherapy, more than 1 month waiting time to begin treatment, and being HR and HER2 positive contributed to a poorer prognosis in MBC patients.

An additional nomogram model 3 was constructed based on the Fine and Gray method to predict the probability of the occurrence of an SPM. Li et al. focused on the SPM on female breast cancer patients and constructed a nomogram to predict the SPM probability of female breast cancer patients (30). A similar study was published by Bao et al. on female breast cancer patients (31). Mellemkjær et al. investigated whether pregnancy near the time of the initial female breast cancer diagnosis would increase the risk of an SPM and obtained a negative result (32). Chen et al. found that germline pathogenic variants in BRCA1, BRCA2, and ERCC2 increased the risk for female breast cancer patients of developing an SPM (33). Nevertheless, no similar research had been published in MBC patients and few studies had focused on the SPM issue in MBC patients. Satram-Hoang et al. found that there is a general tendency towards higher risks of SPM among younger men compared to older men but did not provide a predictive model (34). Hung et al. found that the risk of SPM was significantly higher for both male and female breast cancer patients compared with the general population (35). In this study, we constructed an available nomogram to predict the SPM probability of MBC patients. There were 36 SPM patients in the development cohort and 24 in the validation (**Table 1**). Nomogram model 3 achieved good performance in the C-index and DCA curve in the development cohort and attained an ordinary score in the validation cohort, which was attributed to the rarity of MBC and the small number of the enrolled SPM patients. However, the present study is still the first research to look into the SPM of MBC patients, and achieved an AUC at 0.934 (**Figure 6B**), which indicated a good predictive value of the predictive model.

A nomogram had been widely used for the prediction of certain clinical outcomes because it is convenient and reliable. In this study, we, for the first time, constructed competing risk nomograms including both the treatment information and the clinicopathological parameters to predict the prognosis of MBC patients and, for the first time, developed a competing risk nomogram to predict the probability of developing an SPM in MBC patients, which was thought to be helpful for both clinicians and the patients to estimate the risk and manage their strategies about treatment and follow-up.

There are some limitations in our study. First, this study was a population-based retrospective study using the SEER Plus database, which had missed some important variables of some of the patients, leading to more than 1,000 MBC patients being excluded because of the incomplete information. Second, some important risk factors for SPM that were rapidly developing or widely used in clinical practice nowadays, such as diet and lifestyle, family history of cancer, oncogene test, radiotherapy or chemotherapy protocols, and the performance of endocrine therapy, targeted therapy, or immunotherapy, were not included in the SEER Plus database. Additionally, MBC is a rare disease, and the SEER Plus database did not involve a larger population worldwide, which had restricted the scale of the present study and might lead to bias. An additional larger study is needed to determine the mechanism of SPM in MBC patients.

## Conclusion

Our study for the first time included the treatment information and clinical parameters needed to construct an external validation competing risk nomogram to predict the survival probability of MBC patients, according to which higher age at diagnosis, higher TMN stage, absence of surgery and radiotherapy, more than 1 month waiting time to begin treatment, and being HR and HER2 positive contributed to a poorer prognosis in MBC patients. This study also, for the first time, constructed a nomogram to predict the probability of developing an SPM in MBC survivors, which was helpful in individual risk estimation, patient follow-up, and counseling in MBC patients.

## Data availability statement

The original contributions presented in the study are included in the article/**Supplementary Material**. Further inquiries can be directed to the corresponding authors.

## Ethics statement

The studies involving human participants were reviewed and approved by the Ethics Committee of Guangzhou Red Cross Hospital of Jinan University. Written informed consent for participation was not required for this study in accordance with the national legislation and the institutional requirements.

## Author contributions

HH, ZL, ZH, and LH performed the study, analyzed the data, prepared figures and/or tables, and authored or reviewed drafts of the paper. WL, GL, and YM conceived and designed the study, performed the study, authored or reviewed drafts of the paper, and approved the final draft. All authors contributed to the article and approved the submitted version.
