# Supplementary figures and images for: Development and validation of nomograms to predict the survival probability and occurrence of a second primary malignancy of male breast cancer patients: a population-based analysis
Source: Front Oncol. 2023 Apr 20;13:1076997. doi: 10.3389/fonc.2023.1076997 (PMC10157191; doi:10.3389/fonc.2023.1076997)

Supplementary Figure 1. Flowchart

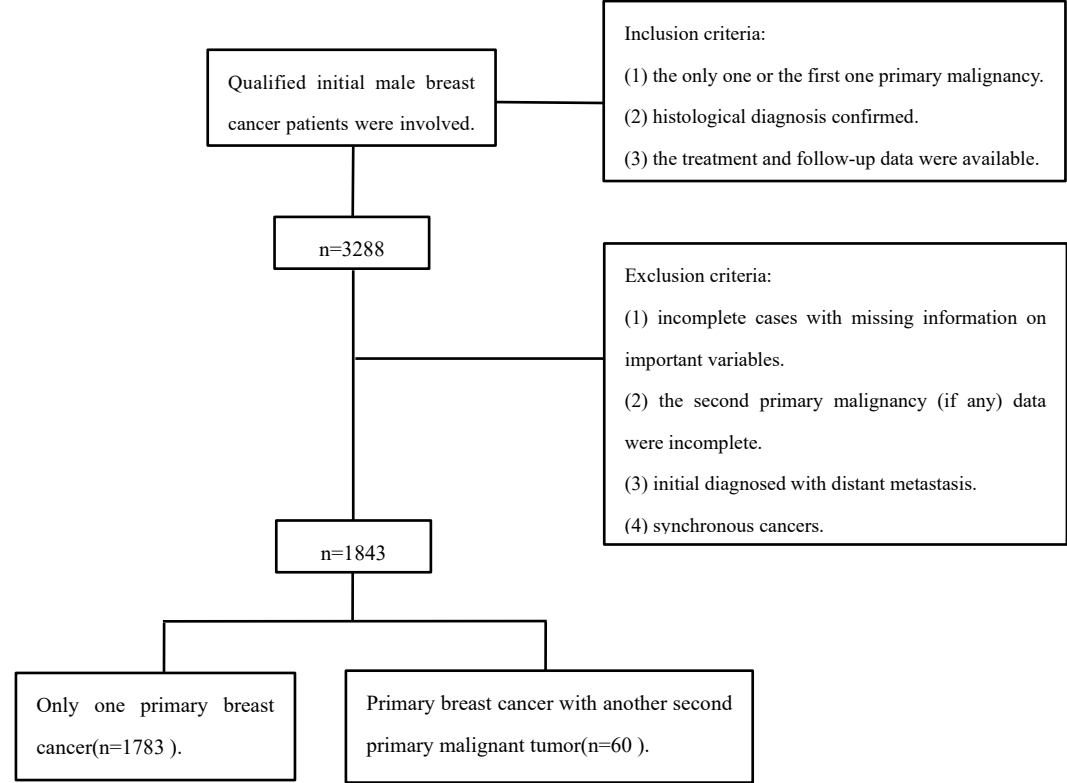

Supplement: Supplementary file 1 [file Image_1.pdf]
