# Supplementary material for: Development and validation of nomograms to predict the survival probability and occurrence of a second primary malignancy of male breast cancer patients: a population-based analysis
Source: Front Oncol. 2023 Apr 20;13:1076997. doi: 10.3389/fonc.2023.1076997 (PMC10157191; doi:10.3389/fonc.2023.1076997)
Supplement: Supplementary file 2 [file Table_1.doc]

**Supplementary Table 1.**

**Detailed point of the variables in nomogram model 1**

| Age | points |
| --- | --- |
| 65-75 | 9 |
| 55-65 | 4 |
| 75-85 | 14 |
| 45-55 | 0 |
| 85+ | 24 |
| <45 | 17 |
|  |  |
| Race | points |
| White | 12 |
| Black | 16 |
| Other | 0 |
|  |  |
| Marital status | points |
| Married | 7 |
| Single | 0 |
| Divorced | 7 |
|  |  |
| Tumor Grade | points |
| Grade II | 53 |
| Grade III | 65 |
| Grade I | 54 |
| Grade IV | 0 |
|  |  |
| Histological type | points |
| Infiltrating duct | 18 |
| Adenocarcinoma | 22 |
| Other | 0 |
|  |  |
| TMN Stage | points |
| I | 55 |
| IIA | 63 |
| IIB | 80 |
| IIIA | 88 |
| IIIC | 100 |
| IIIB | 71 |
| 0 | 0 |
|  |  |
| Surgery performed | points |
| Yes | 0 |
| No | 23 |
|  |  |
| Radiotherapy performed | points |
| No | 3 |
| Yes | 0 |
|  |  |
| Chemotherapy performed | points |
| No | 0 |
| Yes | 3 |
|  |  |
| Months to begin treatment | points |
| ≤ 1 month | 0 |
| ＞ 1 month | 3 |
|  |  |
| HR status | points |
| Positive | 3 |
| Negative | 0 |
|  |  |
| HER2 status | points |
| Negative | 0 |
| Positive | 8 |
|  |  |
| Total Points | Probability for 5-year survival |
| 244 | 0.2 |
| 238 | 0.3 |
| 233 | 0.4 |
| 228 | 0.5 |
| 222 | 0.6 |
| 215 | 0.7 |
| 205 | 0.8 |
| 191 | 0.9 |
|  |  |
| Total Points | Probability for 8-year survival |
| 240 | 0.1 |
| 233 | 0.2 |
| 227 | 0.3 |
| 222 | 0.4 |
| 217 | 0.5 |
| 211 | 0.6 |
| 204 | 0.7 |
| 194 | 0.8 |
| 180 | 0.9 |
|  |  |
| Total Points | Probability for 10-year survival |
| 235 | 0.1 |
| 228 | 0.2 |
| 223 | 0.3 |
| 217 | 0.4 |
| 212 | 0.5 |
| 206 | 0.6 |
| 199 | 0.7 |
| 190 | 0.8 |
| 175 | 0.9 |
