# Supplementary material for: Development and validation of nomograms to predict the survival probability and occurrence of a second primary malignancy of male breast cancer patients: a population-based analysis
Source: Front Oncol. 2023 Apr 20;13:1076997. doi: 10.3389/fonc.2023.1076997 (PMC10157191; doi:10.3389/fonc.2023.1076997)
Supplement: Supplementary file 3 [file Table_2.doc]

**Supplementary Table 2.**

**Detailed point of the variables in nomogram model 2**

| Age | points |
| --- | --- |
| 65-75 | 7 |
| 55-65 | 3 |
| 75-85 | 11 |
| 45-55 | 0 |
| 85+ | 19 |
| <45 | 14 |
|  |  |
| Tumor Grade | points |
| Grade II | 59 |
| Grade III | 72 |
| Grade I | 59 |
| Grade IV | 0 |
|  |  |
| TMN Stage | points |
| I | 55 |
| IIA | 65 |
| IIB | 82 |
| IIIA | 90 |
| IIIC | 100 |
| IIIB | 72 |
| 0 | 0 |
|  |  |
| Surgery performed | points |
| Yes | 0 |
| No | 25 |
|  |  |
| Total Points | Probability for 5-year survival |
| 209 | 0.2 |
| 203 | 0.3 |
| 198 | 0.4 |
| 192 | 0.5 |
| 185 | 0.6 |
| 178 | 0.7 |
| 168 | 0.8 |
| 152 | 0.9 |
|  |  |
| Total Points | Probability for 8-year survival |
| 205 | 0.1 |
| 198 | 0.2 |
| 192 | 0.3 |
| 186 | 0.4 |
| 180 | 0.5 |
| 174 | 0.6 |
| 166 | 0.7 |
| 156 | 0.8 |
| 141 | 0.9 |
|  |  |
| Total Points | Probability for 10-year survival |
| 200 | 0.1 |
| 193 | 0.2 |
| 187 | 0.3 |
| 181 | 0.4 |
| 175 | 0.5 |
| 169 | 0.6 |
| 161 | 0.7 |
| 151 | 0.8 |
| 136 | 0.9 |
