# Supplementary material for: Development and validation of nomograms to predict the survival probability and occurrence of a second primary malignancy of male breast cancer patients: a population-based analysis
Source: Front Oncol. 2023 Apr 20;13:1076997. doi: 10.3389/fonc.2023.1076997 (PMC10157191; doi:10.3389/fonc.2023.1076997)
Supplement: Supplementary file 4 [file Table_3.doc]

**Supplementary Table 3.**

**Detailed point of the variables in nomogram model 3**

| Age | points |
| --- | --- |
| 65-75 | 95 |
| 55-65 | 76 |
| 75-85 | 84 |
| 45-55 | 81 |
| 85+ | 0 |
| <45 | 11 |
|  |  |
| Race | points |
| White | 99 |
| Black | 0 |
| Other | 21 |
|  |  |
| Marital status | points |
| Married | 0 |
| Single | 5 |
| Divorced | 29 |
|  |  |
| Tumor Grade | points |
| Grade II | 88 |
| Grade III | 0 |
| Grade I | 95 |
| Grade IV | 27 |
|  |  |
| Histological type | points |
| Infiltrating duct | 1 |
| Adenocarcinoma | 18 |
| Other | 0 |
|  |  |
| TMN Stage | points |
| I | 71 |
| IIA | 70 |
| IIB | 81 |
| IIIA | 100 |
| IIIC | 0 |
| IIIB | 94 |
| 0 | 36 |
|  |  |
| Surgery performed | points |
| Yes | 78 |
| No | 0 |
|  |  |
| Radiotherapy performed | points |
| No | 13 |
| Yes | 0 |
|  |  |
| Chemotherapy performed | points |
| No | 0 |
| Yes | 16 |
|  |  |
| Months to begin treatment | points |
| ≤ 1 month | 0 |
| ＞ 1 month | 22 |
|  |  |
| HR status | points |
| Positive | 0 |
| Negative | 1 |
|  |  |
| HER2 status | points |
| Negative | 77 |
| Positive | 0 |
|  |  |
| Total Points | 10-year probability to suffer a SPM |
| 585 | 0.1 |
| 582 | 0.2 |
| 579 | 0.3 |
| 577 | 0.4 |
| 575 | 0.5 |
| 572 | 0.6 |
| 569 | 0.7 |
| 564 | 0.8 |
| 558 | 0.9 |
